# Validation of Foot Angular Measurements Using Intraoperative Simulated Weightbearing Fluoroscopic Images

**DOI:** 10.1177/24730114261417690

**Published:** 2026-02-12

**Authors:** Troye J. Joseph, Nikiforos P. Saragas, Michael de Buys, Paulo N. F. Ferrao

**Affiliations:** 1 Division of Orthopaedic Surgery, School of Clinical Medicine, Faculty of Health Sciences, University of the Witswatersrand, Parktown, Johannesburg, South Africa; 2 Orthopaedic Foot and Ankle Unit, Linksfield Hospital, Johannesburg, South Africa

**Keywords:** simulated weightbearing imaging, foot angular measurements, foot deformity correction, weightbearing radiographs, intraoperative fluoroscopy

## Abstract

**Background:**

Weightbearing foot and ankle radiographs are essential because skeletal geometry changes under load. Radiographic measurements, which guide management decisions, have been validated using weightbearing radiographs. Simulation of weightbearing intraoperatively would be ideal and may improve the accuracy of deformity correction in foot and ankle surgery. This study aims to validate the accuracy of angular measurements on intraoperative simulated weightbearing fluoroscopic foot images.

**Methods:**

A prospective study of 50 patients with a mean age of 51.3 years undergoing elective foot surgery at a single institution was performed. A simulation of weightbearing was performed intraoperatively and fluoroscopic anteroposterior and lateral images were obtained. Six angular measurements were performed on the standard preoperative weightbearing radiographs and compared to the intraoperative simulated weightbearing fluoroscopic images, by 4 researchers at 2 intervals.

**Results:**

The mean differences for the hallux valgus angle (HVA), intermetatarsal angle (IMA), interphalangeal angle (IPA), 4-5 intermetatarsal angle (4-5 IMA), calcaneal pitch (CP), and talocalcaneal angle (TCA) were +0.02, −1.79, +1.13, −0.01, +4.80, and −1.41 degrees, respectively. Of the anteroposterior and lateral measurements, the HVA, IPA, 4-5 IMA, and TCA showed no statistically significant mean difference (paired *t* test), and inter- and intraobserver reliability was good to excellent. The IMA and CP showed a mean difference that was statistically significant; however, this mean difference was clinically negligible (IMA: −1.79 ± 1.68 degrees; CP: 4.8 ± 3.4 degrees). A good inter- and intraobserver reliability was found between researchers. Regression analyses showed strong correlations for the HVA, IMA, 4-5 IMA, and CP and fair correlations for the TCA and IPA.

**Conclusion:**

The study suggests the technique we use for intraoperative simulated weightbearing fluoroscopic imaging correlates with standard preoperative weightbearing foot radiographs and may facilitate a more accurate, real time assessment of alignment during foot deformity correction surgery.

**Level of Evidence:**

Level II, diagnostic.

## Introduction

Foot and ankle pathologies are some of the most common conditions presenting to orthopaedic surgeons.^1-3^ In assessing these conditions, weightbearing radiographs are paramount as the skeletal geometry of the foot changes during the stance phase and often symptoms are experienced with loading of the foot.^4^ Boszczyk et al^5^ highlighted the clinical importance of weightbearing radiographs by showing significant differences between treatment decisions regarding patients with hallux valgus deformities, based on measurements obtained on weightbearing versus non-weightbearing radiographs. Furthermore, common radiographic measurements used in practice have been validated on weightbearing radiographs^4-15^ and standardised methods of evaluating these measurements are described on weightbearing radiographs, summarised by Coughlin et al^16^ as part of the committee of the American Orthopaedic Foot & Ankle Society (AOFAS).

In a prospective study, Gutteck et al^17^ compared the intraoperative fluoroscopy images of 31 hallux valgus correction patients, which were non-weightbearing, to the postoperative weightbearing radiographs. They showed that the measured intraoperative angles and the top range values of these angles were notably lower when compared to the angles measured on the post-operative weightbearing views. Taking this into consideration, in foot and ankle surgery, a simulation of a weightbearing load intraoperatively would be ideal and may improve the accuracy of deformity corrections.

Ahluwalia et al in their study compared intraoperative simulated weightbearing fluoroscopic anteroposterior (AP) foot images and immediate postoperative non-weightbearing radiographs to standard postoperative weightbearing radiographs at 6 weeks and final postoperative weightbearing radiographs. Their results suggested that an intraoperative simulated weightbearing image gives a closer approximation to the weightbearing view at 6 weeks compared with non-weightbearing postoperative radiographs, most particularly with the hallux valgus angle.^18^

Boffeli and Mahoney prospectively looked at intraoperative simulated weightbearing fluoroscopic lateral images during the Lapidus procedure. They highlighted that the assessment of the sagittal plane alignment with fluoroscopic imaging is difficult and is limited due to the usual fluoroscopic images being non-weightbearing. They evaluated 48 patients who underwent a Lapidus procedure with a simulated weightbearing lateral fluoroscopic imaging technique. They compared these images with the 10-week postoperative weightbearing radiographs, by assessing sagittal plane alignment of the first metatarsal (MT). All comparisons showed direct correlations between the simulated weightbearing fluoroscopic images and the postoperative radiographs. As a result, the authors suggested that intraoperative simulated weightbearing lateral fluoroscopic imaging is an accurate representation of the final postoperative result in terms of the first MT sagittal plane position after the Lapidus procedure.^19^ Similarly, in a later study, Boffeli and Duelfer^20^ found that intraoperative simulated weightbearing lateral images were accurate in predicting the final postoperative sagittal plane alignment of the foot during reconstructive surgery for flatfoot deformity correction.

When obtaining weightbearing foot and ankle radiographs, it is presumed that approximately half of a patient’s weight is bore on each lower limb. Theoretically so, one would assume that the same amount of force would be required to represent a simulation of weightbearing intraoperatively. However, Shelton et al looked at foot geometry changes with varying percentage weightbearing in healthy individuals. They demonstrated that once a threshold of 25% of a patient’s body weight is reached, a further increase in body weight did not further alter the radiographic geometry of the foot. The authors thus concluded that provided at least 25% body weight is applied on the foot and ankle, the radiographs obtained are no different compared to normal weightbearing foot and ankle radiographs.^21^

Considering this, it may be possible to obtain a true representative of weightbearing changes intraoperatively with a simulation of weightbearing and allow for more accurate deformity corrections. Many studies have compared the differences between preoperative non-weightbearing and weightbearing radiographs, but further research is needed regarding intraoperative simulated weightbearing imaging. This study aims to validate the accuracy of intraoperative simulated weightbearing fluoroscopic images by comparing various foot angular measurements on these images to standard preoperative weightbearing radiographs.

## Methods

This prospective study included 50 consecutive patients who underwent elective foot surgery at a single institution. The inclusion criteria for this study were patients undergoing foot surgery who had routine preoperative standard weightbearing foot radiographs performed to international standards and who required intraoperative fluoroscopic imaging. Patients who had undergone previous foot surgery were excluded.

Ethics approval was obtained from the local institution’s Human Research Ethics Committee (Medical). Statistical power was calculated to determine the minimum number of subjects necessary to evaluate accuracy within 1 degree of each of the angles measured and a margin of error of 0.05 with a 95% confidence interval (CI). The minimum number of subjects needed was calculated to be 44. A total of 50 patients (37 female, 13 male), with a mean age of 51.3 (range: 12-77) years, met the inclusion criteria for the study.

**Figure 1.**
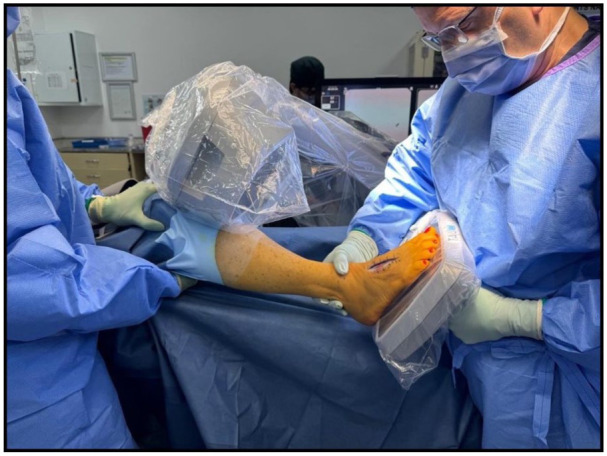
The anteroposterior (AP) image obtained by placing the patient’s foot flat on a sterilely draped mini C-arm image intensifier’s flat detector, with the ankle and knee flexed to 90 degrees, while applying maximal pressure on the limb perpendicular to the foot. With rotational support and counterpressure provided by the assistant.

**Figure 2.**
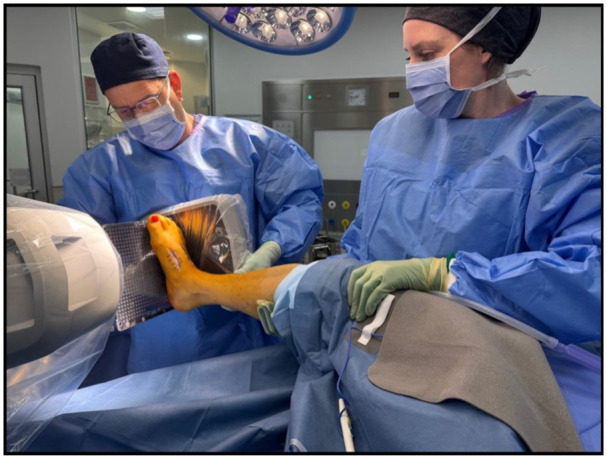
The lateral view obtained using a sterile radiolucent plastic board positioned beneath the foot at 90 degrees to the sterilely draped mini C-arm image intensifier with the knee flexed to 90 degrees, with rotational control and counterpressure provided by the assistant, while the foot is loaded by the surgeon’s hand pressing up against the plastic board with the ankle in a neutral position.

The simulated intraoperative weightbearing fluoroscopic foot images were obtained in theatre by a single surgeon with the patient under general anaesthesia. The AP image was obtained by placing the patient’s foot flat on a sterilely draped mini C-arm image intensifier’s flat detector, with the knee flexed to 90 degrees (the knee supported by the assistant to minimise rotation and translation), while applying maximal pressure on the limb perpendicular to the foot (Figure 1). The lateral view was obtained using a sterile radiolucent plastic board positioned beneath the foot, in a neutral position, at 90 degrees to the sterilely draped mini C-arm image intensifier. The patient’s knee was flexed to 90 degrees with rotational control and counterpressure provided by the assistant, while the foot was loaded by the surgeon’s hand pressing up against the plastic board with the ankle in a neutral position (Figure 2). Images were saved to the patient’s digital medical records, and all radiographs and images were analysed using the hospital’s digital Picture Archiving and Communication System (PACS), version 5.0.2.1 (Infinitt Healthcare Co.).

**Figure 3.**
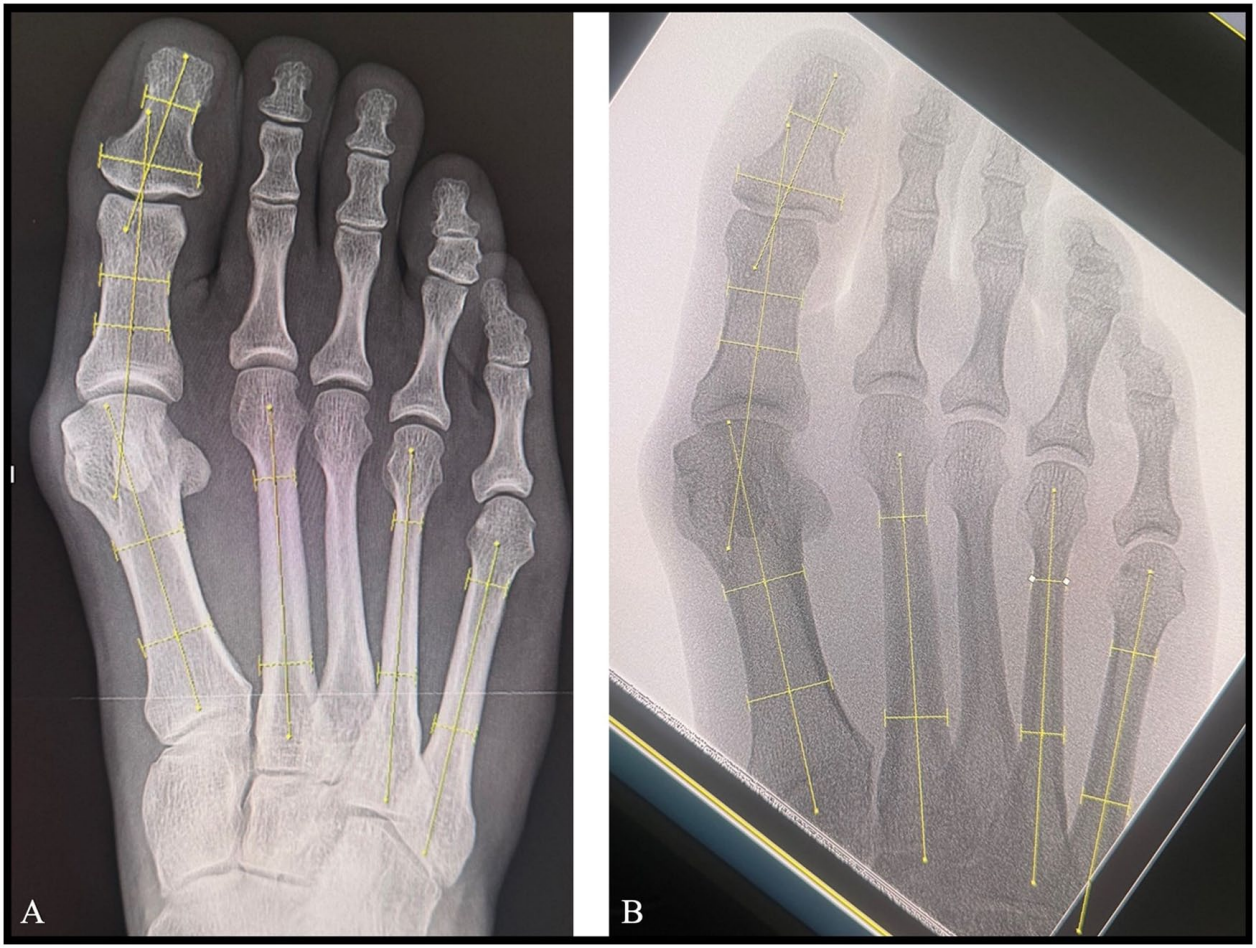
The hallux valgus angle (HVA), intermetatarsal angle (IMA), interphalangeal angle (IPA) and the 4-5 intermetatarsal angle (4-5 IMA) measurements performed (A) on each of patients’ standard preoperative weightbearing anteroposterior (AP) foot radiographs and (B) on the AP intraoperative simulated weightbearing fluoroscopic images.

**Figure 4.**
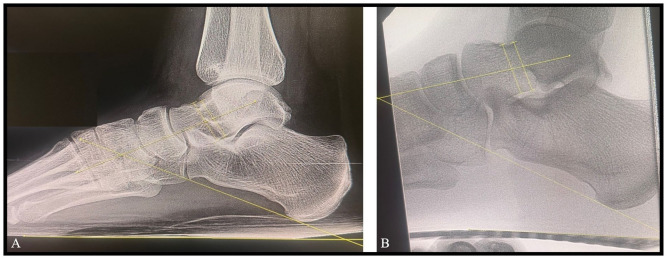
The calcaneal pitch (CP) and talocalcaneal angle (TCA) measurements performed (A) on each of patients’ standard preoperative weightbearing lateral foot radiographs and (B) on the lateral intraoperative simulated weightbearing fluoroscopic images.

To validate the accuracy of intraoperative simulated weightbearing fluoroscopic images, angular measurements were performed on each of the patients’ standard preoperative weightbearing foot radiographs and then performed on the intraoperative simulated weightbearing fluoroscopic images. These measurements were done by 2 certified senior foot and ankle surgeons, 1 junior certified foot and ankle surgeon, and 1 orthopaedic surgery registrar on 2 separate occasions at least 6 weeks apart. All angles were measured on digital images in accordance with the standardised AOFAS guideline and rounded to the nearest 1 decimal place.^16^ The hallux valgus angle (HVA), intermetatarsal angle (IMA), interphalangeal angle (IPA), and the 4-5 intermetatarsal angle (4-5 IMA) were measured on the AP images (Figure 3). Likewise, the calcaneal pitch (CP) and talocalcaneal angle (TCA) were measured on the lateral images (Figure 4). Demographic data of the patients including patient age, gender, and foot side was recorded (Table 1) along with the angular measurements.

**Table 1.** Patient Characteristics.

	Data
Sample size (patients)	50
Gender	
Female	37
Male	13
Average age, y	51.3 (12-77)
Affected side	
Right	28
Left	22

Statistical analysis of the angular measurements was performed. The mean, median, and interquartile ranges (IQRs) were calculated for the complete data set. Intraobserver reliability was assessed using intraclass correlation coefficients for each of the angles measured. The interobserver reliability was also determined using intraclass correlation coefficients of the mean value of each angle measured. A regression analysis was performed to determine correlation between the measurements of each angle on the standard preoperative weightbearing foot radiographs and the intraoperative simulated weightbearing fluoroscopic images. The analysis included calculating the *R*^2^ value for each angle and performing a paired *t* test on the mean differences for each angle measured, wherein a *P* value <.05 was considered to be statistically significant. A Shapiro Wilk test was performed to assess if the data was normally distributed.

## Results

The mean differences (Table 2) found on the AP intraoperative simulated weightbearing fluoroscopic images and the standard preoperative weightbearing foot radiographs for the HVA, IMA, IPA, and 4-5 IMA were +0.02, −1.79, +1.13, and −0.01 degrees, respectively. On the lateral images and radiographs the mean difference between the CP was +4.80 degrees and the TCA was −1.41 degrees. Of the AP and lateral measurements the HVA, IPA, 4-5 IMA, and TCA showed no statistically significant mean difference using a paired *t* test with a *P* <.05 (Table 2). The IMA and CP showed a mean difference that was statistically significant. However, this mean difference was less than 5 degrees for the CP and less than 2 degrees for the IMA. Using a Shapiro-Wilk test, two-thirds of the measurements performed showed a normal distribution.

**Table 2.** Mean Differences Between Intraoperative Simulated Weightbearing Fluoroscopic Images and the Standard Preoperative Weightbearing Foot Radiographs.

Angle	Mean Difference ± SD (degrees)	Paired *t* Test, *P* Value	Statistical Significance (*P* Value < .05)
HVA	0.02 ± 4.2	.9740	Not statistically significant
IMA	−1.79 ± 1.68	<.001	Statistically significant
IPA	1.13 ± 4.3	.0663	Not statistically significant
4-5 IMA	−0.01 ± 1.3	.9642	Not statistically significant
CP	4.8 ± 3.4	<.001	Statistically significant
TCA	−1.41 ± 5.2	.0610	Not statistically significant

Abbreviations: CP, calcaneal pitch; HVA, hallux valgus angle; IMA, intermetatarsal angle; IPA, interphalangeal angle; SD, standard deviation; TCA, talocalcaneal angle.

The interobserver reliability was shown to be good (Table 3). The HVA demonstrated an excellent intraclass correlation coefficient (ICC) at 0.9149, good reliability was also shown for IMA, IPA, 4-5 IMA, and CP, whereas the TCA showed a moderate interobserver reliability. The ICCs for all angles measured demonstrated low *P* values (*P* < .05). An intraobserver reliability was also performed using an intraclass correlation coefficient between researchers. All researchers had a good intraobserver reliability for the HVA, IMA, 4-5 IMA, whereas 3 of the 4 researchers were found to have a good intraobserver reliability for the IPA, CP, and TCA measurements.

**Table 3.** Interobserver ICC Between Intraoperative Simulated Weightbearing Fluoroscopic Images and the Standard Preoperative Weightbearing Foot Radiograph Measurements.

	Preoperative (degrees)		Intraoperative (degrees)		Interobserver ICC	Intracorrelation *P* Value
Angle	Mean (SD)	Median (IQR)	Mean (SD)	Median (IQR)		
HVA	17.7 (10.1)	15.5 (9.6-25.1)	17.7 (8.5)	16.7 (10.2-24.2)	0.9149	<.0001
IMA	11.8 (3.4)	11.3 (9.7-13.0)	10.0 (8.5)	16.7 (10.2-24.2)	0.8810	<.0001
IPA	12.1 (5.2)	12.2 (9.1-14.3)	13.2 (5.8)	12.9 (9.8-16.3)	0.7048	<.0001
4-5 IMA	7.6 (2.9)	7.5 (5.9-9.6)	7.6 (2.9)	7.3 (6.5-9.6)	0.8920	<.0001
CP	15.1 (5.6)	15.4 (12.9-17.9)	19.9 (6.0)	7.3 (16.9-22.9)	0.8309	<.0001
TCA	47.6 (6.2)	47.6 (42.6-51.5)	46.2 (5.8)	47.2 (42.1-49.6)	0.6275	<.0001

Abbreviations: CP, calcaneal pitch; HVA, hallux valgus angle; ICC, intraclass correlation coefficient; IMA, intermetatarsal angle; IPA, interphalangeal angle; IQR, interquartile range; SD, standard deviation; TCA, talocalcaneal angle.

**Figure 5.**
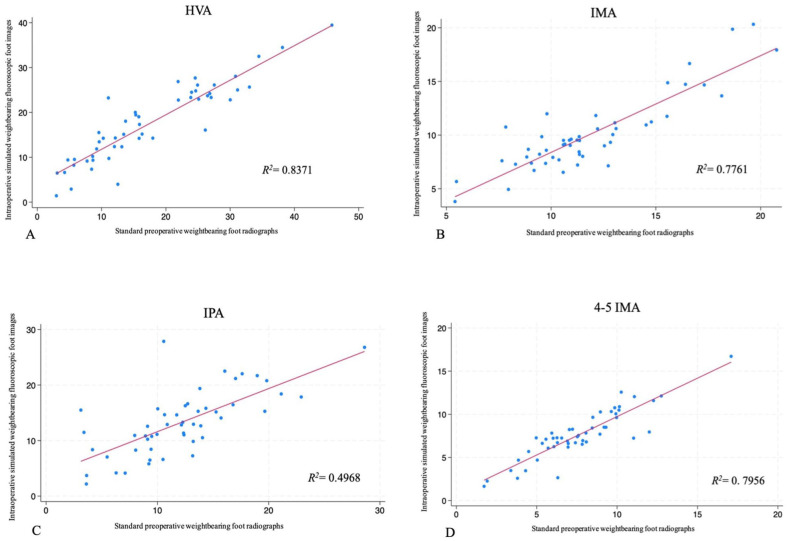
Regression analyses for AP angular measurements. (A) Hallux valgus angle (HVA). (B) Intermetatarsal angle (IMA). (C) Interphalangeal angle (IPA). (D) 4-5 intermetatarsal angle (4-5 IMA).

**Figure 6.**
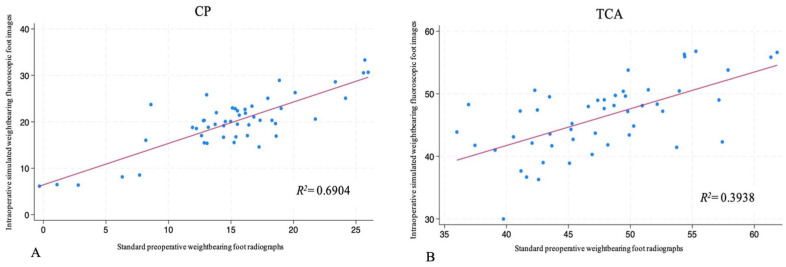
Regression analyses for Lateral angular measurements. (A) Calcaneal pitch (CP). (B) Talocalcaneal angle (TCA).

A regression analysis was performed to assess the correlation between the angles measured on the standard preoperative weightbearing foot radiographs compared to the intraoperative simulated weightbearing fluoroscopic foot images (Table 4). A very strong correlation was found for the HVA (*R*^2^ = 0.8371), and strong correlations (*R*^2^ > 0.6) were found for the IMA, 4-5 IMA (Figure 5), and CP (Figure 6). The analysis, however, found a moderate correlation (*R*^2^ = 0.4968) for the IPA (Figure 5) and a moderate to weak correlation (*R*^2^ = 0.3938) for the TCA (Figure 6).

**Table 4.** Regression Analysis Between Standard Preoperative Weightbearing Foot Radiographs Measurements Compared With Intraoperative Simulated Weightbearing Fluoroscopic Images.

Angle	*R*^2^ Value	*P* Value
HVA	0.8371	<.001
IMA	0.7761	<.001
IPA	0.4968	<.001
4-5 IMA	0.7956	<.001
CP	0.6904	<.001
TCA	0.3938	<.001

Abbreviations: CP, calcaneal pitch; HVA, hallux valgus angle; IMA, intermetatarsal angle; IPA, interphalangeal angle; TCA, talocalcaneal angle.

## Discussion

The results of this study support the premise that simulation of weightbearing intraoperatively gives a close representation of the geometric changes of the foot that occur with standard weightbearing. We found the mean differences between standard preoperative weightbearing foot radiographs compared to the intraoperative simulated weightbearing fluoroscopic foot images to be marginal. Moreover, all the mean differences were within 2 degrees, except for the CP, which was within 5 degrees, and all the mean differences for each angle measured were within a standard deviation (SD) of 5.2 degrees or less. Similarly to Boffeli and Mahoney^19^ we found the use of a lightweight radiolucent plastic board combined with a standardised protocol to be an effective, accurate and reproducible means for obtaining simulated weightbearing fluoroscopic images.

The regression analysis performed revealed a correlation between the standard preoperative weightbearing foot radiographs compared to the intraoperative simulated weightbearing fluoroscopic foot images wherein a strong correlation was found for 5 of the 7 angles measured. The HVA was the most accurate of angles measured with a mean difference of 0.02 degrees and a paired *t* test *P* value of .9740, indicating the mean difference was not statistically significant. The HVA also demonstrated the strongest correlation using a regression analysis with a *R*^2^ value of 0.8371. A similar result was calculated for the 4-5 IMA, showing a strong correlation with a *R*^2^ value of 0.7956. The IPA was shown to have a moderate correlation having the lowest *R*^2^ value (*R*^2^ = 0.4968) of the angles measured on AP radiographs and images. Although the IPA correlation was not shown to be strong with the regression analysis, the mean difference between the intraoperative and preoperative images was +1.13 degrees, and this difference was found not to be statistically significant.

Our findings, however, did suggest a weak correlation between the lateral preoperative radiographs and intraoperative images for the TCA. However, the mean difference between the measurements on the lateral preoperative radiographs and images was not statistically significant and the simulated intraoperative weightbearing TCA mean difference was less than 2 degrees of the preoperative radiographs. On the contrary, the CP had a mean difference of +4.8 degrees and even though this difference was shown to be statistically significant, the CP on the lateral preoperative radiographs and intraoperative images was shown to have a strong correlation (*R*^2^ = 0.6904) after the regression analysis was performed.

Concerning the IMA, the mean difference found between the intraoperative images and the preoperative radiographs was −1.79 degrees. Although this difference was also found to be statistically significant, it was less than 2 degrees and a further regression analysis showed a strong correlation between the 2 measurements with a *R*^2^ value of 0.7761. Saltzman et al^22^ in their study demonstrated that the IMA could be measured with a 95% CI within 4 degrees, and the HVA within 6 degrees. Similarly, Coughlin et al^16^ and Coughlin and Freund^23^ found that IMA measurements were reproducible within 5 degrees or less in 96.7% of cases. Although both studies primarily address the intraobserver reliability of these measurements, they collectively suggest that a variability of up to 5 degrees is likely acceptable in clinical practice. In the current study, the mean difference variability among all the angles ranged from 0.01 to 4.8 (±1.3-5.2) degrees, which is within the acceptable norm for clinical practice.

The interobserver reliability analysis we performed was good between the 4 researchers. Of the angles measured, the HVA demonstrated an excellent reliability and good reliability was found for IMA, IPA, 4-5 IMA, and CP. The TCA had the lowest ICC but showed a moderate interobserver reliability. The ICCs performed all showed low *P* values (*P* < .0001), suggesting that the observed correlations were statistically significant. The intraobserver reliability was found to be good between 3 of the 4 researchers across all angles measured. However, 1 researcher showed good reliability across the majority of measured angles except for the IPA, CP and TCA measurements, which showed modest reliability.

We acknowledged limitations that are evident with the present study.

First, the use of a mini C-arm poses a difficulty in obtaining a full lateral intraoperative image depending on the detector size. Multiple images are thus required for different measurements. Second, the amount of pressure that was applied to obtain a simulation of weightbearing was not measured and inevitably this will lead to some variability in the amount of force applied. Third, rotation was minimised by assistant support but not rigidly controlled. However, keeping in mind that only a theoretical threshold of 25% of a patient’s body weight is needed to obtain the same geometric change that would occur in the foot with normal weightbearing,^21^ this may not be a significant limitation and our results are consistent with previous studies assessing the accuracy of simulated intraoperative weightbearing fluoroscopic images with a similar technique.^18-20^

## Conclusion

The study suggests the technique we use for simulated intraoperative weightbearing fluoroscopic imaging correlates with standard preoperative weightbearing foot radiographs with good intra- and interobserver reliability. This method is simple and may allow for a more accurate, real-time assessment of alignment during foot deformity correction intraoperatively and could help anticipate postoperative weightbearing radiographic alignment.

## Supplemental Material

sj-pdf-1-fao-10.1177_24730114261417690 – Supplemental material for Validation of Foot Angular Measurements Using Intraoperative Simulated Weightbearing Fluoroscopic Images Supplemental material, sj-pdf-1-fao-10.1177_24730114261417690 for Validation of Foot Angular Measurements Using Intraoperative Simulated Weightbearing Fluoroscopic Images by Troye J. Joseph, Nikiforos P. Saragas, Michael de Buys and Paulo N. F. Ferrao in Foot & Ankle Orthopaedics
